# Validation of AI-based markerless gait event detection during perturbed walking using smartphone videos from two camera perspectives

**DOI:** 10.3389/fspor.2026.1902010

**Published:** 2026-08-04

**Authors:** Valerie Graf, Vanessa Haug, Daniel Seebacher, Manuel Stein, Michael Denkinger, Markus Gruber, Neltje Piro, Michael Munz, Tim Fleiner, Michael Schwenk

**Affiliations:** 1 Department of Sport Science, Human Performance Research Centre, University of Konstanz, Konstanz, Germany; 2 Institute for Geriatric Research, Ulm University, Ulm, Germany; 3 Geriatric Center, Agaplesion Bethesda Clinic, Ulm, Germany; 4 Subsequent GmbH, Konstanz, Germany; 5 Institute of Computer Science, Ulm University of Applied Sciences, Ulm, Germany; 6 Institute of Medical Engineering and Mechatronics, Ulm University of Applied Sciences, Ulm, Germany

**Keywords:** artificial intelligence, markerless gait event detection, perturbation treadmill, reactive balance, temporal gait analysis

## Abstract

**Background:**

Perturbation-based balance training (PBT) requires objective quantification of reactive stepping responses, yet gait event detection during perturbed walking remains methodologically challenging. Markerless smartphone-based gait analysis offers a scalable alternative to laboratory motion capture systems, but its robustness under perturbation-induced gait irregularities is insufficiently validated. This pilot study including five participants explored the validity of the SMARTGAIT markerless motion analysis system during normal and perturbed treadmill walking in older adults and investigated whether perturbation-specific model retraining could improve gait event detection.

**Methods:**

Five geriatric participants (mean age 83 ± 4.47 years; 40% female) walked on a perturbation treadmill while eight unexpected perturbations in both mediolateral and anteroposterior displacement were administered. Smartphone videos from frontal and diagonal perspectives were recorded and gait events (initial contact, final contact) were manually annotated as ground truth. Model performance was evaluated using F1 score, precision, and recall with a ± 33 ms tolerance window. Retraining was conducted using five-fold cross-validation on perturbed segments.

**Results:**

Across 2,477 annotated gait events (2,106 normal; 371 perturbed), initial detection performance was lower during perturbed walking (median F1 score: frontal 0.49; diagonal 0.88) compared to normal walking (median F1 score: frontal 0.68; diagonal 0.96), with large effect sizes despite non-significant *p*-values (frontal: *p* = 0.10; diagonal: *p* = 0.06). This decrease was mainly recall-driven, whereas precision remained high. No statistically significant differences were found between diagonal and frontal camera perspectives; however, large effect sizes (normal walking: r = 0.87; perturbed walking: r = 1.00) suggest that camera perspective may influence detection accuracy. Retraining was associated with higher perturbed gait event detection in the frontal perspective (F1 score: 0.49 → 0.76, *p* = 0.06), mainly accompanied by higher recall (0.34 → 0.64, *p* = 0.06), but showed no change in the diagonal perspective (F1 score: 0.88 → 0.88; *p* = 0.18).

**Conclusion:**

This pilot study provides proof of concept for the application of smartphone-based markerless gait event detection during perturbation-induced walking. The findings further support perturbation-specific model retraining as a feasible strategy for adapting AI-based gait analysis to challenging movement conditions. Larger validation studies are required to confirm these preliminary findings before clinical implementation.

## 1 Introduction

Falls in older adults represent a major public health concern and are a leading cause of injury, loss of independence, and healthcare utilization. Approximately 30% of individuals over 65 years’ experience at least one fall annually, frequently resulting in fractures, traumatic brain injuries, or long-term functional decline (1). Impaired reactive balance control during unexpected mechanical disturbances while walking is a central contributor to fall risk (2, 3).

Reactive stepping responses are essential for restoring stability following sudden perturbations. Perturbation-Based Balance Training (PBT) has therefore emerged as a task-specific intervention designed to improve compensatory stepping capacity under destabilizing conditions (4). Randomized controlled trials and systematic reviews demonstrate that PBT can reduce fall risk in older adults and neurological populations (5–7). Specialized perturbation treadmills allow standardized and reproducible anteroposterior and mediolateral disturbances, enabling controlled assessment and training of reactive balance responses (8, 9).

Despite increasing clinical implementation of PBT, objective quantification of reactive stepping remains methodologically limited. Clinical observation lacks temporal precision and inter-rater reliability (10). Laboratory-based marker-based motion capture systems provide high temporal accuracy but require specialized infrastructure, are resource-intensive, and are therefore not transferable for routine clinical application (11, 12). This gap between laboratory-grade measurement and clinical feasibility limits scalable implementation of evidence-based PBT assessment (13).

Markerless motion analysis using RGB cameras and deep learning-based pose estimation has emerged as a promising alternative. Recent systematic reviews indicate that markerless systems can achieve comparable accuracy to marker-based systems for spatiotemporal gait parameters during normal walking (12). Convolutional neural network-based pose estimation frameworks have demonstrated promising validity in clinical gait analysis under steady-state conditions (14–17). Smartphone-based approaches are particularly attractive for clinical translation due to low cost, mobility, and minimal setup requirements.

However, most validation studies focus on unperturbed walking characterized by symmetric and periodic gait cycles. Perturbation-induced gait responses differ substantially from steady-state walking. Compensatory reactions frequently involve asymmetries, altered foot strike patterns, shortened or aborted steps, and exaggerated upper-limb movements (18, 19). Such irregular and non-periodic movement patterns challenge algorithmic assumptions underlying many gait event detection models and may impair generalization performance, particularly when models are trained predominantly on steady-state gait data (20). As a result, evidence obtained during steady-state walking cannot be readily transferred to perturbation-based balance training scenarios. Whether markerless gait analysis systems remain valid under such highly variable and reactive gait conditions is currently unclear.

Therefore, validating markerless AI-based gait event detection under perturbed treadmill conditions is essential before clinical integration. In addition, practical factors such as camera perspective may influence detection robustness due to occlusion effects and differences in foot-ground visibility. Furthermore, targeted retraining strategies may improve model robustness when confronted with distribution shifts induced by perturbations.

The SMARTGAIT system combines a 3D Pose Estimation Model (3D-PEM) with a Gait Cycle Estimation Model (GCEM) to derive temporal gait events from monocular smartphone video. Previous validation demonstrated feasibility and validity during non-perturbed walking in neurological populations (21). Its performance under perturbation-based balance training, however, has not yet been systematically examined yet.

The objective of this study was to validate the SMARTGAIT motion analysis approach during perturbation-based treadmill walking in older adults at increased risk of falls using frame-accurate manual annotations as the reference standard. In addition, we examined the influence of camera perspective and the potential benefit of perturbation-specific model retraining. Specifically, three predefined hypotheses within a clinical perturbation paradigm were tested: (1) detection performance, quantified by the F1 score, would be lower during perturbed compared to normal walking; (2) camera perspective (frontal vs. diagonal) would significantly influence classification performance; and (3) targeted retraining on perturbed segments would improve F1 scores during perturbed walking.

## 2 Materials and methods

### 2.1 Study design

This cross-sectional pilot study evaluated the performance of the markerless AI-based gait event detection system SMARTGAIT against frame-accurate manual annotations during normal and perturbed treadmill walking in older adults. Event-level validation of initial contact (IC) and final contact (FC) was performed, including comparisons between walking conditions, camera perspectives, and model performance before and after targeted retraining using cross-validation. Each participant served as their own reference within a within-subject validation framework.

### 2.2 Participants

Five participants (three male, two female) were randomly selected from a dataset of 40 geriatric patients who had previously completed PBT at the falls clinic at AGAPLESION BETHESDA KLINIK ULM. The mean age was 83 ± 4.47 years (range 76–88 years). The cohort consisted of older adults with clinically documented balance impairments, and the majority reported at least one fall within the previous 12 months. Clinical characteristics are presented in Table 1.

**Table 1:** Participant characteristics.

Variables	ID1	ID2	ID3	ID4	ID5
Age	85	84	88	82	76
Gender	m	m	m	f	f
CHARMI®, score	9	10	10	10	10
CFS, score	5	2	3	3	2
SPPB, score	6	9	12	5	4
5CR, sec	18.26	15.77	9.36	30.90	31.53
4-MWT, sec	5.10	3.79	4.60	5.42	6.92
Falls past 12 month	yes	no	yes	yes	yes
Neurologic disease	no	no	no	Poly- neuro- pathy	no

Participant information: **m** = male, **f** = female, **CHARMI** = Charité Mobility Index: 0 = complete immobility, 10 = complete mobility; **CFS** = Clinical Frailty Scale: 1 = very active/fit, 8 = extremely frail/9 = life expectancy <6 months; **SPPB** = Short Physical Performance Battery: 0–4 = reduced mobility, 5–8 = moderate limitations in mobility, 9–12 = good mobility and independence, < 9 = increased falls risk; **5CR** = Five Chair Rise Test; **4-MWT** = 4-Meter Walk Test; **TUG** = Timed Up and Go Test; Falls last 12 months, ≥ 2 Falls in General; Results presented in “seconds”.

Participants were eligible if they were aged 65 years or older, achieved a Charité Mobility Index [CHARMI® (22),] score of at least 6, and were able to walk independently without assistive devices within the room. Exclusion criteria included a palliative condition with life expectancy below three months, inability to provide informed consent, inability to walk at least two minutes on a treadmill, uncontrolled cardiovascular, metabolic, or psychiatric conditions, severe osteoporosis, recent fractures, open or chronic wounds, surgical intervention within the preceding eight weeks, and body weight exceeding 135 kg due to device limitations.

The study was conducted in accordance with the Declaration of Helsinki and approved by the Ethics Committee of the University Hospital Ulm (reference number 73/24). Written informed consent was obtained from all participants prior to participation.

### 2.3 Experimental setup

Participants walked on a perturbation treadmill (BalanceTutor™, MediTouch Ltd., Israel), which enables programmable anteroposterior and mediolateral perturbations via a movable force plate platform. During each session, participants completed six walking bouts of approximately two minutes each. A total of eight perturbations were automatically triggered per session at varying intensities ranging from 1 to 25 [Attachment 1 and Supplementary Material S.5. in (23)]. Perturbations included sudden accelerations, brief belt stops, and lateral displacements to both sides (Appendix 1). Participants were not informed about the timing of perturbations to ensure validity of reactive stepping responses.

**Figure 1:**
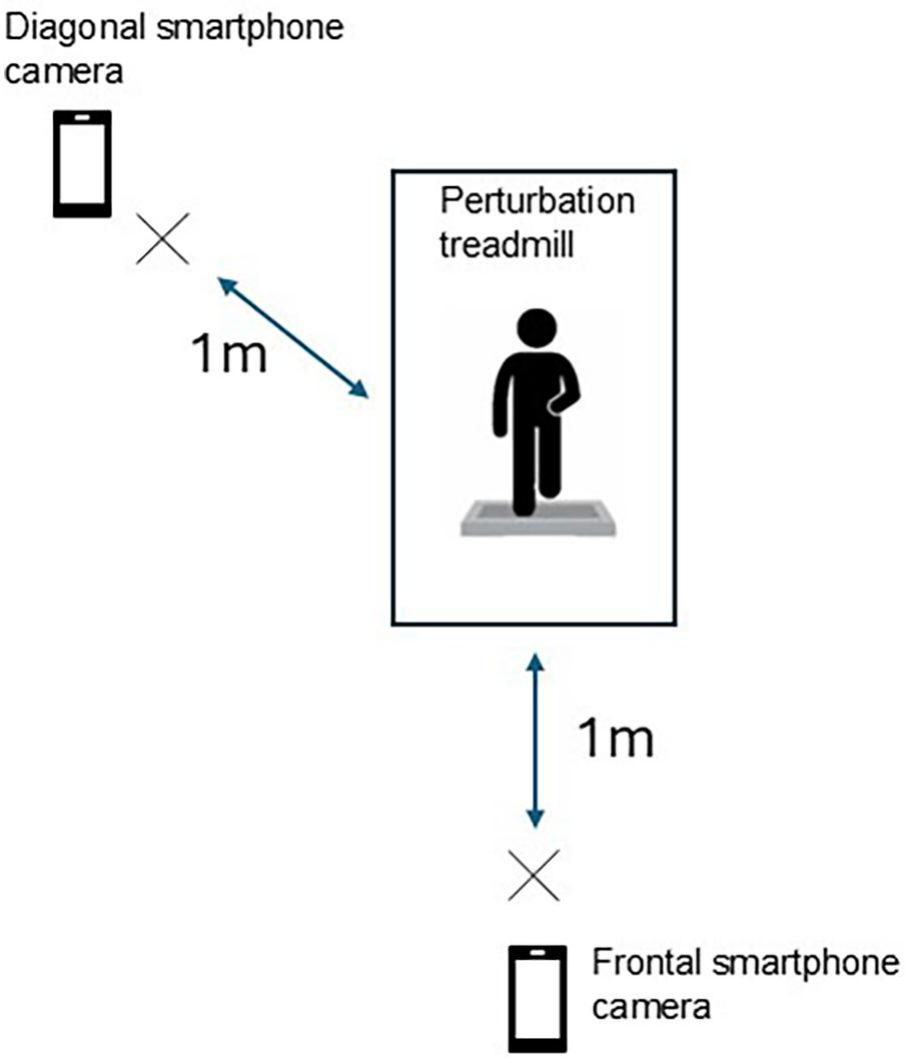
Experimental setup showing the perturbation treadmill and smartphone camera placements. A frontal smartphone camera was positioned 1 m in front of the treadmill, and a diagonal smartphone camera was positioned 1 m laterally to the treadmill to capture gait from two perspectives.

Gait was recorded simultaneously using two smartphone cameras (Apple iPhone SE, 3rd generation, 2022) at a frame rate of 33 frames per second. One camera was positioned one meter in front of the treadmill (frontal perspective), and the second camera was positioned one meter laterally (diagonal perspective). Cameras were mounted on tripods to ensure stability. No strict calibration procedure was applied, reflecting realistic clinical implementation conditions. Participants were secured with a safety harness connected to the treadmill throughout testing (Figure 1).

### 2.4 Manual annotation

In the present study, five video recordings were manually annotated by a trained human observer using Label Studio (version 1.13.1). Annotation was conducted exclusively using the diagonal camera perspective to ensure optimal visibility of foot-ground interactions and to maintain consistency across recordings. Manually annotated data are widely regarded as the gold standard reference for validatingautomated motion detection algorithms (24). For gait event detection, frame-by-frame manual annotation is commonly used as a reference standard because it enables direct temporal identification of initial and final foot contact events. The present study therefore focused on agreement between SMARTGAIT-derived gait events and expert-observed gait events rather than on comparison of full kinematic parameters.

For each recording, initial contact (IC) and final contact (FC) of the left and right foot with the treadmill belt were identified and marked (Attachment 2). The gait cycle was analysed frame by frame, and contact events were determined based on visual inspection of foot-ground interaction. Frame-level precision was applied throughout the annotation process.

Video duration ranged from two to three minutes per recording, corresponding to approximately 3,000 to 4,000 frames per video at a frame rate of 33 frames per second. The manually annotated contact events served as the ground truth for evaluating SMARTGAIT performance by direct temporal comparison with automated model predictions.

In addition to foot contact events, the onset of each perturbation was annotated. Furthermore, a subsequent two-second recovery interval following each perturbation was marked. Accordingly, the annotation protocol included systematic labelling of both perturbation onset and the corresponding two-second recovery phase to enable differentiation between steady-state and perturbation-related gait segments. All initial and final contacts occurring within the subsequent two-second recovery interval were classified as perturbed walking.

### 2.5 SMARTGAIT markerless motion tracking

The SMARTGAIT project is a collaborative initiative between Subsequent GmbH, the Lurija Institute for Rehabilitation Science and Health Research at Kliniken Schmieder, and the Movement Science group at the University of Konstanz. The SMARTGAIT system was developed to provide a clinically applicable, markerless gait analysis framework capable of extracting relevant temporal and spatial gait parameters from monocular RGB video. Its clinical feasibility and validity for neurological populations during non-perturbed walking have previously been demonstrated (25).

SMARTGAIT is based on deep learning–driven pose estimation and consists of two in-house developed components: a 3D Pose Estimation Model (3D-PEM) and a Gait Cycle Estimation Model (GCEM). The 3D-PEM employs a convolutional neural network to reconstruct three-dimensional skeletal key points from standard RGB video recordings, including smartphone footage. In contrast to depth-camera systems, which are constrained by limited measurement ranges, distance-dependent resolution loss, and infrared artifacts, RGB-based monocular systems offer greater flexibility and clinical scalability. Furthermore, multi-camera marker-based systems remain cost-intensive and infrastructure-dependent, limiting routine clinical use (11, 12).

The SMARTGAIT 3D reconstruction approach builds upon established pose estimation frameworks, conceptually comparable to algorithms such as OpenPose (14), which have demonstrated high agreement with marker-based motion capture systems (15). The implemented 3D-PEM processes video data in real time and generates a full-body skeletal representation. The skeleton data were represented as a 78-dimensional feature vector comprising the x-, y-, and z-coordinates of 24 key points and six joint angles at the hip, knee, and ankle joints used to compute gait parameters.

The second component, the Gait Cycle Estimation Model (GCEM), operates on the temporal sequence of pose features generated by the 3D-PEM. Specifically, the input consists of a tensor containing n consecutive video frames, where each frame is represented by the 78-dimensional feature vector described above. These sequential features are processed using a bidirectional long short-term memory (BiLSTM) network to estimate frame-wise probabilities of initial contact (IC) and final contact (FC) events separately for the left and right foot. To determine the final gait event timings, peak detection is applied to each resulting one-dimensional probability sequence by identifying local maxima relative to neighbouring values. The detected peaks are interpreted as the predicted IC and FC events and exported as frame indices for subsequent comparison with frame-accurate manual annotations.

The 3D Pose Estimation Model (3D-PEM) was trained on a heterogeneous dataset comprising proprietary recordings, synthetically generated data, and publicly available datasets, including MS COCO. The Gait Cycle Estimation Model (GCEM) was subsequently trained using the 3D-PEM output generated from approximately 300 individuals exhibiting both healthy and pathological gait patterns during steady-state walking. Notably, no perturbation treadmill data were included in the original training data. Consequently, detection of gait events during perturbation-induced stepping responses represents a novel application scenario characterized by increased variability and non-periodic movement patterns.

In the present study, smartphone videos recorded during perturbation-based balance training served as input to the 3D-PEM. The resulting skeletal representations were processed by the GCEM to extract IC and FC events. These model-derived gait events were subsequently compared to frame-accurate manual annotations within the validation framework described above.

To enable quantitative comparison between the diagonal and frontal camera perspectives, a temporal synchronization of both video streams was performed. Manual gait event annotations were originally created for the diagonal perspective and therefore required alignment with the frontal recordings prior to statistical analysis.

A global temporal offset between the two perspectives was estimated using four foot contact events that had been manually annotated in the frontal recordings. These events were matched to the corresponding contacts in the diagonal annotations, and the temporal difference between both recordings was calculated.

The resulting offset was subsequently applied to all diagonal annotations, thereby generating temporally aligned event timestamps for the frontal perspective. This synchronization enabled the derivation of comparable temporal gait parameters and performance metrics across both camera views. Following synchronization, the aligned recordings were visually inspected side-by-side and a random sample of gait events was cross-checked across both camera perspectives. No evidence of relevant temporal drift, frame drops, or synchronization errors was observed.

### 2.6 Event matching and validation metrics

The performance of the SMARTGAIT system was evaluated using an event-based validation framework by comparing model-derived gait events with frame-accurate manual annotations. In line with established validation procedures in machine learning (26) classification outcomes were derived from confusion matrices constructed at the event level.

Predicted initial contact (IC) and final contact (FC) events were temporally matched with annotated ground truth events using a predefined tolerance window of ±33 milliseconds. For performance evaluation, IC and FC events were pooled to calculate a single confusion matrix and overall F1 score. However, event matching was performed separately for each event type; thus, an IC prediction could only be matched to an annotated IC event and an FC prediction only to an annotated FC event. This tolerance corresponds to one video frame at the acquisition rate of 33 frames per second and is consistent with thresholds applied in previous gait event detection studies (19). A predicted event was classified as a true positive (TP) if it occurred within this temporal window relative to a manually annotated event of the same type. Predictions without a corresponding annotation within the tolerance window were classified as false positives (FP), whereas annotated events without a matching prediction were classified as false negatives (FN).

Based on the confusion matrix, precision and recall were calculated. Precision was defined as the proportion of correctly predicted events among all predicted events, while recall represented the proportion of correctly detected annotated events. The primary performance metric was the F1 score, defined as the harmonic mean of precision and recall. The F1 score was selected because it provides a balanced evaluation of detection performance and is particularly robust under conditions of class imbalance (27, 28). Given the substantially lower number of perturbed compared to normal gait events, reliance on overall accuracy would have biased performance estimates.

F1 scores were computed separately for normal walking and perturbed walking, for each camera perspective (frontal and diagonal), and for each participant. Median F1 scores across participants were used for group-level reporting. Median precision and recall values were computed to assess whether the F1 scores were primarily driven by precision or recall.

### 2.7 Model retraining and cross-validation

To investigate whether targeted adaptation improves robustness under perturbation-induced distribution shift, the SMARTGAIT model was retrained exclusively on perturbed gait segments. A five-fold cross-validation procedure (K = 5) was implemented to ensure subject-independent evaluation and to reduce the risk of overfitting. This form of cross-validation is referred to as Leave-One-Subject-Out Cross-Validation (LOSOCV).

In each fold, data from four participants were used for training, while data from the remaining participant served as the independent test set. This leave-one-subject-out structure within the five-fold framework ensured that evaluation was performed on unseen individuals. Training samples consisted of randomly extracted windows of 128 consecutive time steps from the training recordings. The perturbation dataset was approximately class-balanced, with comparable numbers of initial and final contacts for both the left and right foot.

Retraining was restricted to the final classification layer of the Gait Cycle Estimation Model, while earlier layers of the pose estimation pipeline remained frozen. This strategy was chosen to preserve previously learned generic movement representations while allowing adaptation of the final decision boundary to perturbation-specific gait characteristics. Such partial retraining approaches are commonly used to mitigate overfitting when adapting deep learning models to smaller domain-specific datasets.

**Figure 2:**
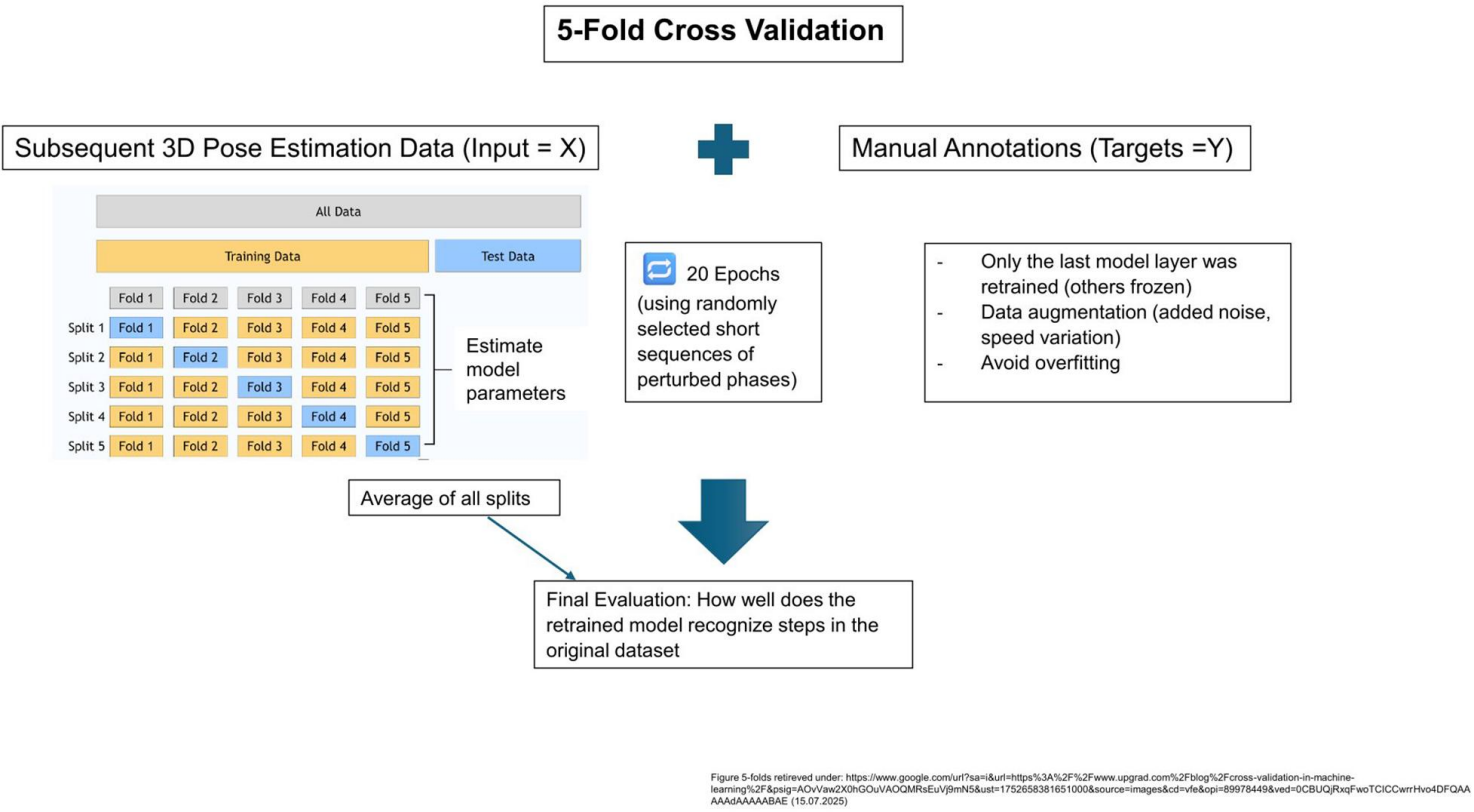
It shows the 5-fold cross-validation procedure for retraining a 3D pose estimation-based step recognition model. The dataset is split into five folds, with four used for training and one for testing in each iteration. Only the final layer is retrained over 20 epochs, while the others remain frozen. Data augmentation is applied to reduce overfitting, and performance is averaged across all splits for the final evaluation.

Training was performed over twenty epochs with a batch size of sixteen samples. A piecewise constant learning rate schedule (PiecewiseConstantDecay) was applied, with learning rates of 1 × 10⁻^3^ for epochs 1–10, 5 × 10⁻⁴ for epochs 11–15, and 1 × 10⁻⁴ for epochs 16–20. These hyperparameters were determined prior to cross-validation and kept constant across all folds. Data augmentation techniques, including random temporal cropping, probabilistic time warping, amplitude scaling, additive Gaussian jitter, and magnitude warping, were applied during training to improve generalization robustness. Time warping was applied jointly to the input signals and target annotations to preserve temporal alignment. Fine-tuning was performed for a fixed 20 epochs without early stopping. No test-fold information was used during hyperparameter selection or model optimization. After completion of cross-validation, post-training performance was evaluated using the identical event-matching criteria and F1-based validation framework described above. Baseline and post-training F1 scores were directly compared (Figure 2).

### 2.8 Statistical analysis

All statistical analyses were conducted using Python (version 3.12) and JASP (version 0.18.1.0). Given the small sample size (*n* = 5) and the non-normal distribution of performance metrics, non-parametric Wilcoxon signed-rank tests were applied for inferential comparisons.

Three primary comparisons were conducted in accordance with the predefined hypotheses. First, F1 scores, precision and recall for normal and perturbed walking were compared to assess performance degradation under perturbation. Second, F1 scores, precision and recall obtained from frontal and diagonal camera perspectives were compared to evaluate the influence of recording perspective. Third, F1 scores, precision and recall before and after retraining were compared to determine the effect of targeted model adaptation on perturbed gait detection.

Effect sizes were reported as rank-biserial correlations (r), which provide an interpretable measure of effect magnitude for paired non-parametric comparisons. In addition, bootstrapped 95% confidence intervals were calculated for median differences where applicable. Statistical significance was defined as *p* < 0.05.

Given the exploratory character of this pilot study and the limited sample size, emphasis was placed on effect sizes and confidence intervals rather than sole reliance on *p*-values.

## 3 Results

### 3.1 Participants characteristics and data overview

Five participants were included in the analysis. The mean age was 83 years (SD = 4.47; range 76–88 years).

On average, participants achieved a Charité Mobility Index (CHARMI®) score of 9.8, a Clinical Frailty Scale (CFS) score of 3.0, and a Timed Up and Go (TUG) performance of 15.69 s, indicating reduced mobility and increased fall risk within a geriatric population (Table 1).

The perturbation protocol was successfully completed by all participants. Video acquisition from both frontal and diagonal perspectives was performed without technical complications.

Across all recordings, a total of 2,477 gait events were manually annotated, comprising 2,106 normal gait events and 371 perturbed gait events. Video duration ranged from 2:14 to 2:35 min per participant, resulting in a maximum difference of 21 s across recordings.

### 3.2 Performance during normal vs. perturbed walking

In accordance with the first hypothesis, detection performance was lower during perturbed compared to normal walking across both camera perspectives. In the frontal perspective, the median F1 score decreased from 0.68 (min–max: 0.06–0.98) during normal walking to 0.49 (min–max: 0.06–0.91) during perturbed walking. In the diagonal perspective, the median F1 score decreased from 0.96 (min–max: 0.81–0.97) during normal walking to 0.88 (min–max: 0.29–0.93) during perturbed walking (Table 2).

**Table 2:** Model performance across walking conditions and camera perspectives.

Perspective and metric	Normal walking	Perturbed walking	Effect size r	Median difference	*P*-value
F1 score Frontal, median (min, max)	0.68 (0.06, 0.98)	0.49 (0.06, 0.91)	1.0	−0.09	0.10
F1 score Diagonal, median (min, max)	0.96 (0.81, 0.97)	0.88 (0.29, 0.93)	1.0	−0.09	0.06
Precision Frontal, median (min, max)	0.94 (0.84, 0.98)	0.94 (0.85, 1.00)	0.0	−0.01	1.00
Precision Diagonal, median (min, max)	0.97 (0.96, 0.99)	0.98 (0.91, 1.00)	0.0	0.01	1.00
Recall Frontal, median (min, max)	0.53 (0.03, 0.98)	0.34 (0.03, 0.83)	1.0	−0.15	0.10
Recall Diagonal, median (min, max)	0.94 (0.68, 0.98)	0.83 (0.17, 0.88)	1.0	−0.15	0.06

The table presents median F1 scores, precision and recall (with minimum and maximum values) for normal and perturbed walking from frontal and diagonal perspectives. It also reports the effect size (r), the median difference, and the corresponding *p*-values for each perspective.

This decrease was primarily driven by reduced recall rather than precision. In the frontal perspective, recall decreased from 0.53 (min–max: 0.03–0.98) to 0.34 (min–max: 0.03–0.83), whereas precision remained high (0.94; min–max: 0.84–0.98 vs. 0.85–1.00). Similarly, in the diagonal perspective, recall decreased from 0.94 (min–max: 0.68–0.98) to 0.83 (min–max: 0.17–0.88), while precision remained high [0.97 (min–max: 0.96–0.99) vs. 0.98 (min–max: 0.91–1.00)].

None of the comparisons reached statistical significance (F1 score: frontal *p* = 0.10, diagonal *p* = 0.06; recall: frontal *p* = 0.10, diagonal *p* = 0.06; precision: frontal *p* = 1.00, diagonal *p* = 1.00). However, large effect sizes for the F1 score (rank-biserial correlation r = 1.00 for both perspectives) indicated a consistent decrease in detection performance during perturbed walking across participants.

### 3.3 Influence of camera perspective

To evaluate the second hypothesis, F1 scores, precision, and recall obtained from frontal and diagonal camera perspectives were compared before retraining.

No statistically significant differences in F1 score were observed between the diagonal and frontal camera perspectives during either normal walking (median: 0.96 [min-max: 0.81–0.97] vs. 0.68 [min-max: 0.06–0.98], *p* = 0.13) or perturbed walking (median: 0.88 [min-max: 0.29–0.93] vs. 0.49 [min-max: 0.06–0.91], *p* = 0.06). However, large effect sizes were observed for both comparisons (normal walking: rank-biserial correlation r = 0.87, 95% CI [0.34, 0.98]; perturbed walking: r = 1.00, 95% CI [1.00, 1.00]), suggesting a consistent directional difference between camera perspectives despite the lack of statistical significance.

Across both walking conditions, precision remained high in both camera perspectives, whereas the lower performance observed in the frontal perspective was primarily attributable to reduced recall.

### 3.4 Effect of targeted retraining

To test the third hypothesis, perturbed gait detection performance before and after retraining was compared.

Before retraining, a total of 216 perturbed gait events were predicted in the diagonal perspective and 119 in the frontal perspective. After retraining, the number of predicted perturbed gait events increased to 268 (diagonal) and 229 (frontal).

In the frontal perspective, the median F1 score increased from 0.49 (min–max: 0.06–0.91) to 0.76 (min–max: 0.49–0.92), corresponding to a median improvement of 0.30 (bootstrapped 95% CI: [0.08, 0.47]). This increase did not reach statistical significance (*p* = 0.06), although a large effect size was observed (rank-biserial correlation r = 1.00). Recall increased from 0.34 (min-max: 0.03–0.83) to 0.64 (min-max: 0.35–0.90), whereas precision remained high (0.94 [min-max: 0.85–1.00] vs. 0.89 [min-max: 0.81–1.00]).

In the diagonal perspective, the median F1 score remained unchanged at 0.88 (pre: min–max 0.29–0.93; post: 0.63–0.96), with a median difference of 0.03 (bootstrapped 95% CI: [−0.08, 0.29]). Recall increased slightly from 0.83 (min-max: 0.17–0.88) to 0.84 (min-max: 0.48–0.94; *p* = 0.06), whereas precision decreased slightly from 0.98 (min-max: 0.91–1.00) to 0.92 (min-max: 0.87–0.98; *p* = 0.10) (Table 3).

**Table 3:** Changes in model performance following retraining during perturbed walking.

Perspective and metric	Perturbed walking, before training	Perturbed walking, after training	Effect size r	Median difference	*P*-value
F1-score Frontal, median (min, max)	0.49 (0.06, 0.91)	0.76 (0.49, 0.92)	−1.00	0.30	0.06
F1-score Diagonal, median (min, max)	0.88 (0.29, 0.93)	0.88 (0.63, 0.96)	−1.00	0.03	0.18
Precision Frontal, median (min, max)	0.94 (0.85, 1.00)	0.89 (0.81, 1.00)	0.40	−0.04	0.58
Precision Diagonal, median (min, max)	0.98 (0.91, 1.00)	0.92 (0.87, 0.98)	1.00	−0.03	0.10
Recall Frontal, median (min, max)	0.34 (0.03, 0.83)	0.64 (0.35, 0.90)	−1.00	0.32	0.06
Recall Diagonal, median (min, max)	0.83 (0.17, 0.88)	0.84 (0.48, 0.94)	−1.00	0.06	0.06

The table presents median F1 scores, precision and recall (minimum–maximum) for perturbed walking in the frontal and diagonal camera perspectives before and after model training. For each perspective, the effect size (rank-biserial correlation, *r*), the median difference (post–pre), and the corresponding *p*-value from Wilcoxon signed-rank tests are reported.

**Figure 3:**
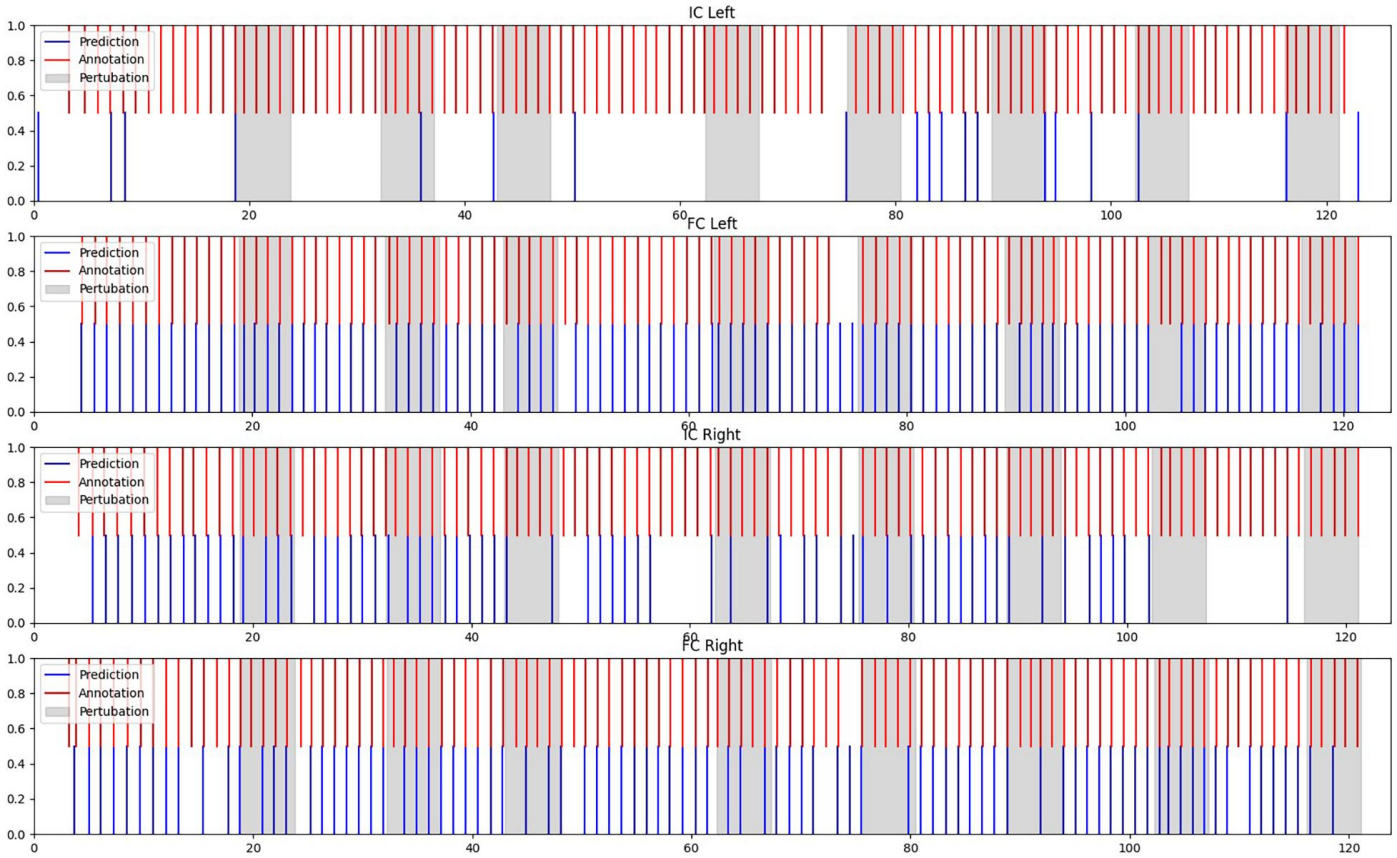
Representative visualization of gait event detection for participant ID4 before model retraining in the frontal perspective. Manually annotated events (red) and SMARTGAIT predictions (blue) are displayed for initial contact (IC) and final contact (FC) of both feet. Grey shaded areas indicate perturbation periods (*n* = 8). After retraining, temporal alignment between predicted and annotated events improved, particularly during perturbation phases.

**Figure 4:**
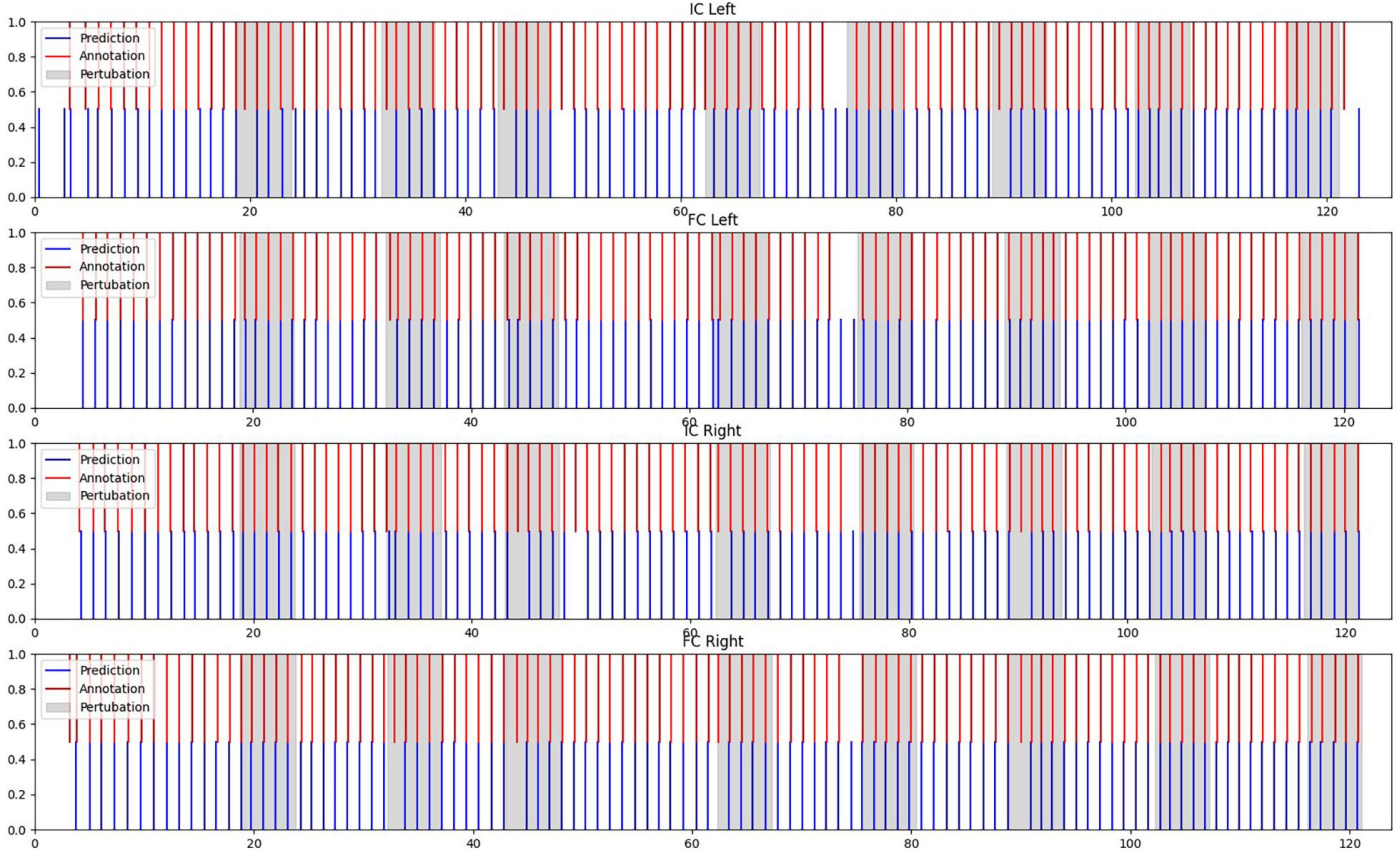
Representative visualization of gait event detection for participant ID4 after model retraining in the frontal perspective. Manually annotated events (red) and SMARTGAIT predictions (blue) are displayed for initial contact (IC) and final contact (FC) of both feet. Grey shaded areas indicate perturbation periods (*n* = 8). After retraining, temporal alignment between predicted and annotated events improved, particularly during perturbation phases.

Representative visualizations of predicted and annotated gait events for participant ID4 are shown in Figures 3, 4. In these plots, manually annotated events are presented in red and SMARTGAIT predictions in blue, with grey shaded areas indicating perturbation periods. Prior to retraining, missed detections were predominantly visible during perturbation phases (Figure 3). Following retraining, temporal alignment between predicted and annotated events increased, particularly in the frontal perspective, corresponding to the quantitative improvement in F1 scores (Figure 4).

## 4 Discussion

The present study evaluated the validity of the SMARTGAIT markerless motion analysis system for detecting gait events during normal and perturbed treadmill walking in older adults undergoing PBT. Three predefined hypotheses were examined. Detection performance tended to be lower during perturbed compared to normal walking. No statistically significant influence of camera perspective on classification performance was observed, although moderate to large effect sizes were present. Targeted retraining showed a trend toward improved perturbed gait event detection, particularly in the frontal perspective; however, these findings should be interpreted cautiously given the pilot nature of the study.

Together, these findings provide proof of concept for the application of smartphone-based markerless gait event detection during perturbation-based treadmill walking while also highlighting current methodological limitations and the need for further validation.

### 4.1 Performance during perturbed walking

In line with the first hypothesis, detection performance was descriptively lower during perturbed walking compared to normal walking across both camera perspectives. Although statistical significance was not reached, effect sizes indicated a consistent reduction across participants. The additional analysis of precision and recall suggests that this performance decrease was mainly recall-driven. Precision remained high and stable across normal and perturbed walking, whereas recall decreased under perturbation conditions. This indicates that the model rarely generated false-positive detections, but was more prone to missed gait events, corresponding to false negatives, during perturbed walking.

This result is biomechanically and methodologically plausible. Perturbation-induced stepping responses are characterized by irregular, non-periodic movement patterns that deviate from steady-state gait (18). Compensatory gait events may involve toe-first contacts, shortened steps, asymmetric loading, or exaggerated upper limb movements, all of which challenge automated detection models trained primarily on regular gait cycles (19). As highlighted by Dumphart and his group (20), deep learning models often rely on learned representations of cyclical movement patterns and may struggle when confronted with atypical or highly variable gait strategies.

The observed variability across participants further supports this interpretation. Detection performance appeared to depend not solely on global physical function but also on the consistency and clarity of movement execution. Participants who demonstrated clearly structured and visible stepping reactions tended to achieve higher F1 scores, whereas irregular or visually ambiguous movement patterns were associated with lower detection performance. This aligns with prior observations that AI-based gait detection can be sensitive to movement variability and visual ambiguity (29).

From a clinical perspective, this is particularly relevant because reactive stepping responses represent the central mechanism targeted by PBT (3, 4). Reliable detection of such responses is essential for objective evaluation of intervention effects (13). The present findings suggest that smartphone-based markerless gait analysis may be feasible in this context, but that perturbation-specific optimization is required to reduce missed events under dynamic walking conditions.

### 4.2 Influence of camera perspective

Overall, the results indicate that camera perspective did not significantly influence gait event detection performance in either walking condition. However, the large effect sizes suggest a consistent directional difference between perspectives. Before retraining, F1 scores were descriptively higher in the diagonal than in the frontal perspective during both normal and perturbed walking. These differences were primarily attributable to recall rather than precision. Precision remained consistently high across perspectives, indicating few false-positive detections, whereas the lower performance observed in the frontal perspective was mainly related to reduced recall, suggesting more missed gait events. This pattern may reflect greater susceptibility of the frontal view to occlusion or reduced visibility of foot-ground contact events.

Thus, the second hypothesis was not statistically supported, although the observed effects indicate that camera perspective may still influence classification performance. During normal walking, higher detection performance in the diagonal view may reflect improved visibility of foot–ground interactions and reduced limb occlusion. In contrast, during perturbed walking, the frontal perspective appeared to perform better, possibly because reactive or lateral compensatory gait events are more clearly captured from the front.

Markerless motion capture systems are known to be sensitive to occlusion, camera angle, and environmental conditions (30). Systematic comparisons between markerless and marker-based systems also emphasize the importance of optimal camera configuration for accurate kinematic reconstruction (12).

Importantly, this finding suggests that technical optimization of recording setup may be as influential as algorithmic refinement. In clinical implementation, camera placement represents a simple and cost-neutral adjustment that may substantially enhance detection robustness.

### 4.3 Effect of targeted retraining

Although retraining did not lead to statistically significant improvements in perturbed gait detection, the results suggest a more consistent detection performance in the frontal perspective, as reflected by the increased F1 scores and the comparatively narrow confidence interval. This may indicate that retraining reduced variability and potential misinterpretations of perturbed gait. This improvement appeared to be mainly driven by increased reall. Precision remained high and changed only slightly. Because perturbation treadmill data were not included in the original training dataset, the baseline model was primarily optimized for steady-state walking. Retraining likely enabled the final classification layer to adapt to perturbation-specific movement characteristics. This interpretation is consistent with findings by Wang et al. (29), who reported that deep learning models benefit from condition-specific training data when detecting gait events under perturbed conditions.

In contrast, detection performance in the diagonal perspective remained largely stable, which may reflect a ceiling effect, as baseline performance was already comparatively high. The limited improvement in this view suggests that the model was already well adapted to the diagonal perspective. Visual inspection of representative plots supports this interpretation, showing improved temporal alignment between predicted and annotated events after retraining. However, the reduced range of F1 scores after retraining suggests that model adaptation may have improved consistency across participants (Table 4), even though the median F1 score changed only minimally. The small increase in recall in the diagonal perspective further supports the interpretation that retraining may help reduce missed events, although this trend requires confirmation in larger samples.

**Table 4:** Per-participant F1 scores across walking conditions, camera perspectives, and model training status.

ID, Perspective, F1-score	Normal walking, before	Normal walking, after	Perturbed walking, before	Perturbed walking, after
ID1 Diagonal	0.96	0.95	0.93	0.96
ID1 Frontal	0.68	0.86	0.59	0.89
ID2 Diagonal	0.97	0.98	0.93	0.93
ID2 Frontal	0.98	0.98	0.91	0.92
ID3 Diagonal	0.81	0.89	0.29	0.63
ID3 Frontal	0.06	0.63	0.06	0.49
ID4 Diagonal	0.97	0.96	0.88	0.88
ID4 Frontal	0.70	0.88	0.49	0.76
ID5 Diagonal	0.90	0.95	0.54	0.70
ID5 Frontal	0.66	0.89	0.24	0.60

Participant-level F1 scores for all walking conditions and camera perspectives are reported in Table 4, illustrating inter-individual variability in detection performance before and after retraining.

Interestingly, the findings also suggest that prior model training on neurological gait patterns (25) may have facilitated detection in participants presenting with pathological gait characteristics. This indicates that exposure to heterogeneous gait data during model development may enhance model robustness when applied to atypical or perturbed gait patterns. Nevertheless, the present results remain exploratory and should be interpreted cautiously due to the small sample size and the lack of statistically significant effects in several comparisons.

### 4.4 Potential for model retraining

The present findings suggest that targeted retraining may represent a promising strategy for improving robustness of AI-based gait event detection under perturbation conditions. Because perturbation treadmill data were not included in the original SMARTGAIT training dataset, baseline performance reflected limited exposure to irregular, non-periodic recovery strategies. Retraining on perturbation-specific segments showed a trend toward improved performance, particularly in the frontal perspective where baseline accuracy was lower.

This finding highlights an important methodological advantage of deep learning–based systems: they are adaptable rather than static. In contrast to rule-based or threshold-based detection algorithms, retrainable neural network models can iteratively refine their decision boundaries when exposed to new movement distributions. Similar improvements following condition-specific retraining have been reported in AI-based gait event detection under perturbed walking conditions (29). In the present study, this trend appeared to be primarily recall-driven, suggesting that retraining may reduce missed gait events rather than substantially increasing false-positive detections.

Perturbation-induced gait responses represent a distribution shift relative to steady-state walking (20). The ability to adapt to such shifts through targeted retraining may therefore be important for ensuring robustness in real-world clinical settings. As additional perturbation-specific data become available, model performance could potentially be systematically enhanced without requiring fundamental architectural changes.

The observed improvement in the frontal perspective suggests that retraining is particularly beneficial when baseline performance is limited. This indicates that model adaptation could reduce sensitivity to suboptimal recording conditions and increase resilience to irregular stepping patterns. Thus, retraining should not be interpreted merely as a corrective measure but a scalable development pathway for continuous performance optimization.

### 4.5 Clinical implications and future scalability

The potential clinical relevance of these findings extends beyond the present pilot study but should be interpreted cautiously given the small sample size. PBT has demonstrated effectiveness in reducing fall risk in older adults (5–7); however, objective and scalable tools for quantifying reactive stepping responses remain limited.

Marker-based motion capture systems provide high biomechanical precision but require specialized infrastructure and are therefore difficult to implement in routine clinical practice (11, 12). Smartphone-based markerless systems may offer a practical and scalable alternative for gait assessment in rehabilitation settings.

The present findings provide proof of concept that markerless gait event detection can be applied during perturbation-induced walking and suggest that perturbation-specific retraining may represent a feasible strategy for adapting AI-based gait analysis to these challenging conditions. Future studies should validate this approach in larger and more heterogeneous cohorts, including different gait impairments, perturbation directions, and perturbation intensities.

If confirmed, such developments could facilitate more objective quantification of reactive stepping responses and support longitudinal assessment of balance recovery during perturbation-based balance training. However, larger validation studies are required before routine clinical implementation.

### 4.6 Study limitations and research perspective

Several limitations must be considered when interpreting the present findings. First, the sample size was small, limiting statistical power and generalizability. Although several effects were large and directionally consistent, the study should be considered exploratory in nature, and the findings should be interpreted as preliminary trends rather than confirmatory evidence.

Second, manual annotation was performed by a single observer without formal assessment of inter-rater or intra-rater reliability. While frame-accurate labelling was applied, the absence of reliability testing may introduce annotation bias.

Consequently, the reported F1 scores, precision and recall values should be interpreted in light of this potential source of observer bias. Future studies should incorporate multiple independent raters and and report inter- and intra-rater reliability metrics.

Furthermore, no marker-based motion capture system (e.g., Vicon) was available for comparison. While frame-by-frame annotation is commonly used as a reference method for gait event detection, future studies should validate the present findings against established laboratory-based gold-standard systems to further confirm the validity of SMARTGAIT under perturbation conditions.

Third, perturbation treadmill data were not included in the original model training dataset. Although targeted retraining showed a trend toward improved robustness, particularly by reducing missed events, the baseline model was optimized for steady-state walking. Inclusion of perturbation-specific gait patterns during initial model development may improve robustness.

Furthermore, markerless systems are inherently sensitive to environmental factors such as lighting conditions, background motion, occlusion, clothing and camera positioning (30). Although real-world clinical conditions were intentionally preserved in this study, controlled comparisons of environmental influences were not performed.

A potential limitation is that residual camera drift and small frame-level synchronization offsets between perspectives may have introduced minor temporal misalignments affecting event timing accuracy. Although no relevant synchronization errors were observed during the visual quality control of the synchronized recordings, small residual temporal offsets cannot be completely excluded.

An additional limitation is the video frame rate of 33 fps, corresponding to a temporal resolution of approximately 30 ms. While this was sufficient to address the primary aims of the present pilot study, higher-frame-rate recordings (e.g., ≥60 Hz) may improve the temporal characterization of rapid perturbation-induced gait responses and should be considered in future validation studies.

Finally, only temporal gait events (initial and final contact) were evaluated. Spatial parameters and dynamic stability metrics were not analysed in the present validation framework. Therefore, the present findings support preliminary feasibility for pilot testing under perturbation conditions, but they do not yet establish clinical utility or readiness for routine implementation.

Future research should include larger and more heterogeneous clinical populations to confirm generalizability across different levels of frailty and neurological impairment. Incorporation of perturbation-specific gait data during initial model training may further improve robustness under non-steady-state conditions.

Systematic investigation of perturbation direction (anteroposterior vs. mediolateral) and intensity effects on detection performance is warranted. Given that perturbation characteristics influence compensatory strategies (18), stratified validation could provide deeper insight into model sensitivity under varying destabilization scenarios.

Beyond event detection, future developments may integrate dynamic stability measures and recovery quality metrics derived from 3D pose trajectories. Such extensions could enhance the clinical relevance of smartphone-based systems for monitoring reactive balance capacity.

Finally, multi-center data acquisition and large-scale retraining pipelines may facilitate continuous model refinement and improved generalization across clinical environments. Establishing standardized data-sharing frameworks could accelerate development of robust and scalable, and clinically applicable markerless gait analysis systems.

## 5 Conclusion

By combining methodological validation with clinically relevant perturbation scenarios, this study provides proof of concept for the application of markerless gait event detection during perturbation-based balance training.

This pilot study suggests that smartphone-based markerless gait event detection using SMARTGAIT is feasible during perturbation-based treadmill walking in older adults. Detection performance decreased under perturbation-induced gait variability, highlighting the challenges of gait event detection under non-steady-state conditions.

Perturbation-specific retraining showed a trend toward improved performance in the lower-performing frontal perspective, suggesting that targeted model adaptation may enhance detection performance under challenging walking conditions.

Although larger validation studies are required before routine clinical implementation, the present findings provide preliminary evidence supporting further development and validation of scalable, AI-based approaches for assessing reactive stepping during perturbation-based balance training.

## Data Availability

The data presented in this study are available on request from the corresponding author due to legal reasons.
