# Supplementary material for: Validation of AI-based markerless gait event detection during perturbed walking using smartphone videos from two camera perspectives
Source: Front Sports Act Living. 2026 Aug 4;8:1902010. doi: 10.3389/fspor.2026.1902010 (PMC13481715; doi:10.3389/fspor.2026.1902010)
Supplement: Supplementary file 1 [file Datasheet1.pdf]

Appendix:

Attachment 1: Perturbation protocol

Level	Perturbation intensity	Perturbation		Time interval between perturbations [sec.]
		Direction	Standing phase	
1	15	FW	R	11
	15	BW	L	11
	10	R	L	8
	15	BW	R	12
	15	FW	L	10
	10	R	R	11
	10	L	R	10
	10	L	L	10
2	15	R	L	12
	20	FW	R	9
	15	L	L	11
	20	FW	L	9
	15	R	R	10
	15	L	R	12
	20	BW	L	8
	20	BW	R	11
3	20	R	L	10
	20	L	R	11
	25	BW	R	12
	25	FW	L	9
	25	FW	R	9
	20	R	R	8
	25	BW	L	12
	20	L	L	10

Attachment 2: Manual annotations in Labelstudio:

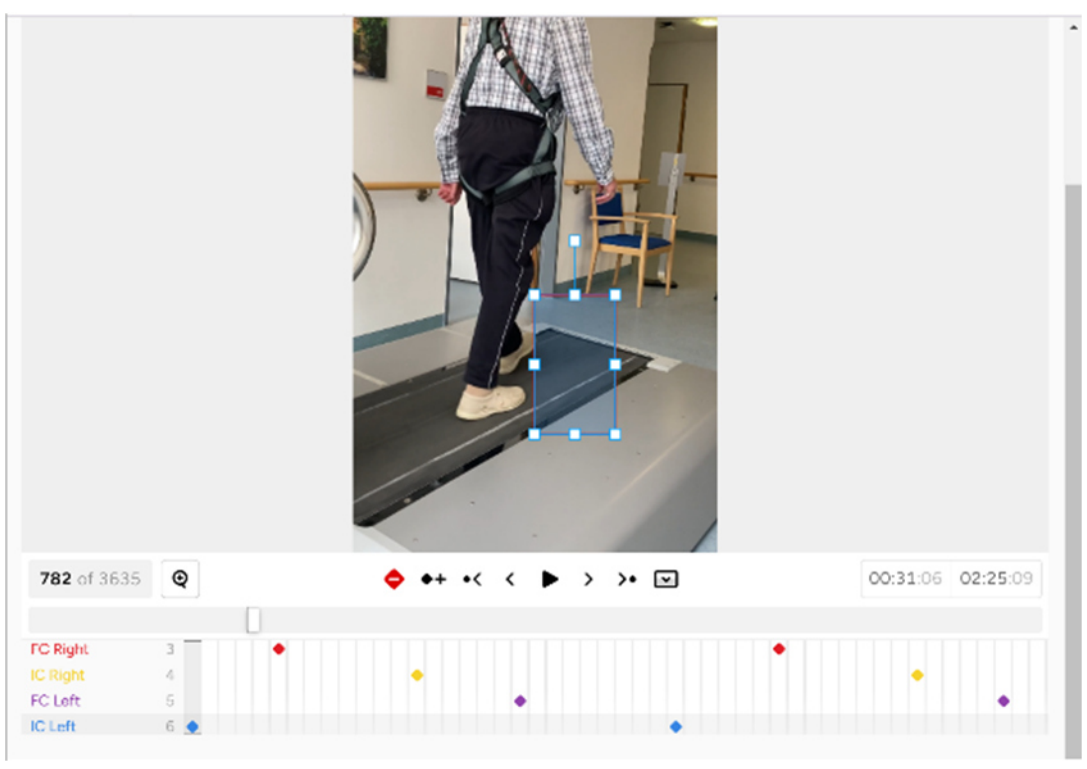

Initial Contact Left

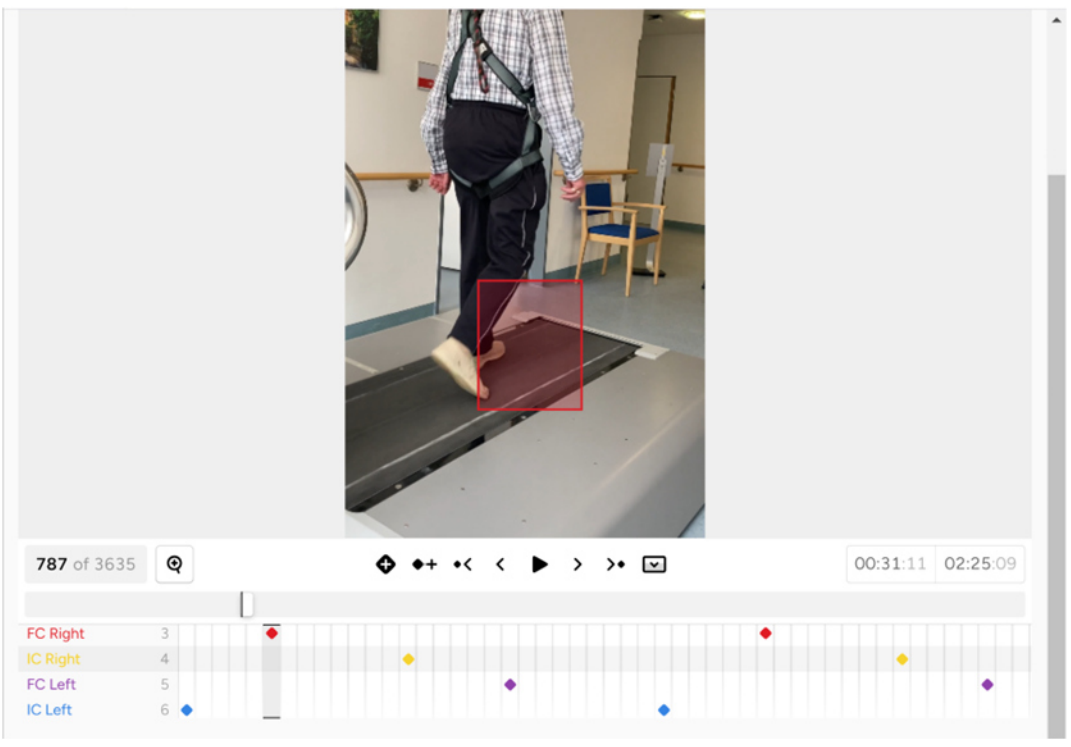

Final Contact Right

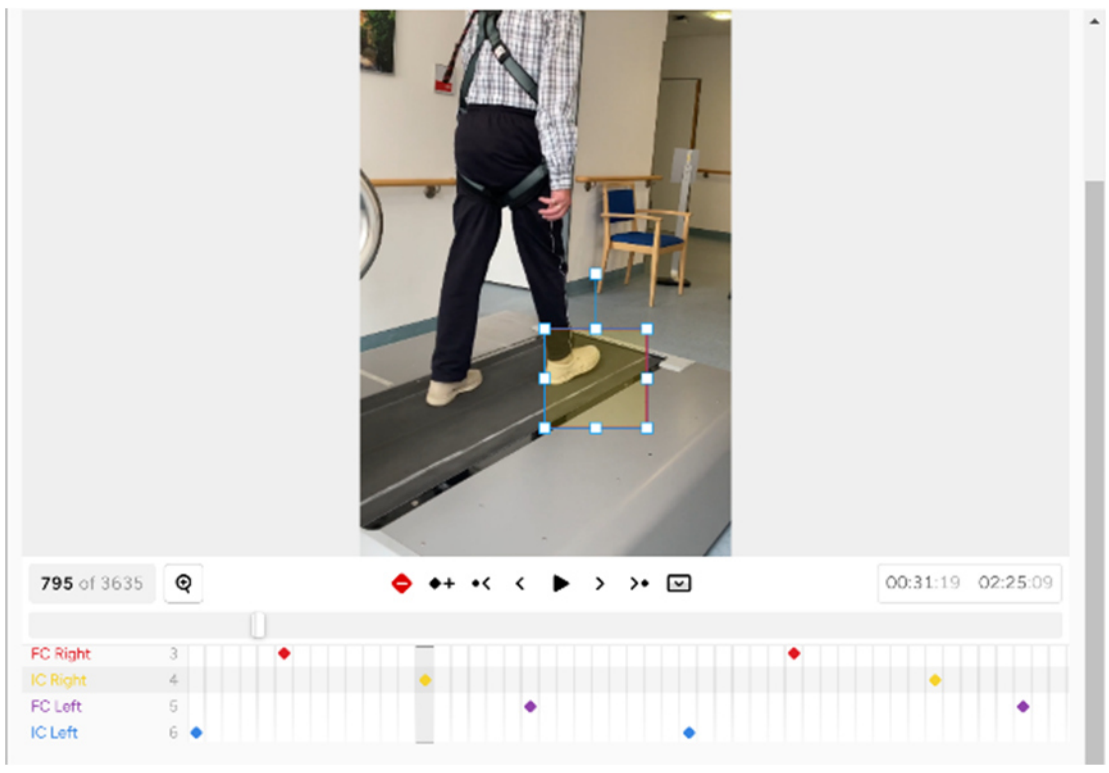

Initial Contact Right

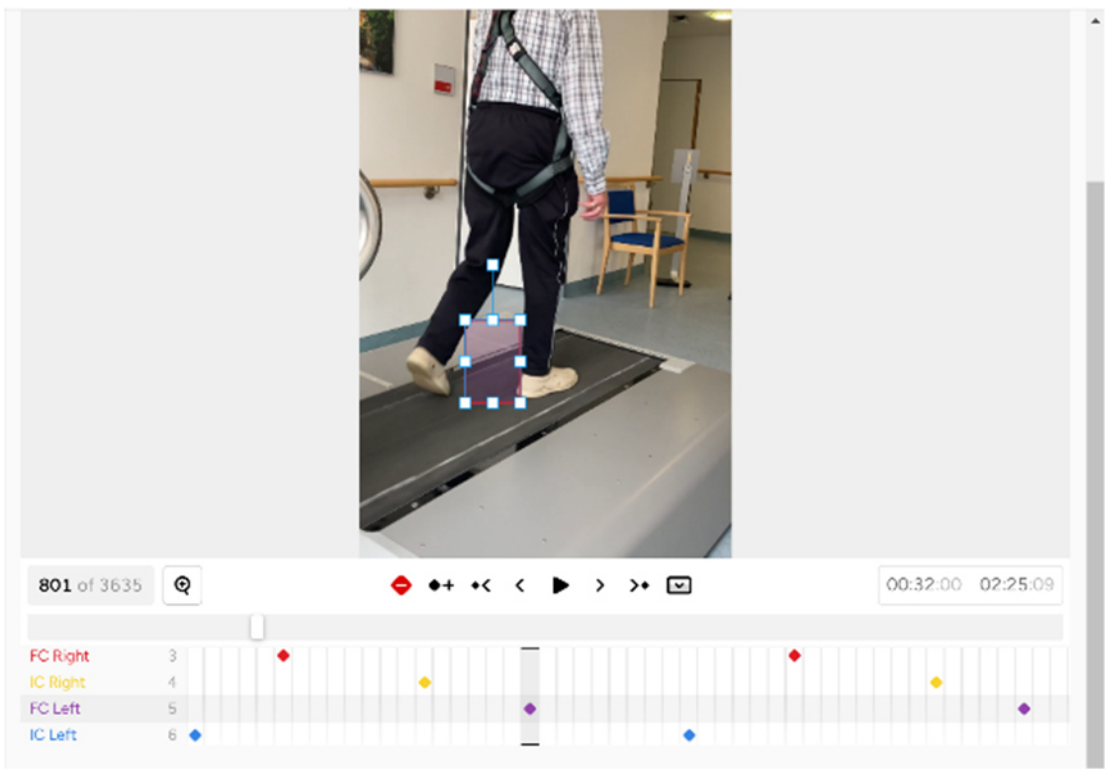

Final Contact Left

Attachment 3: Annotation and Predictions of Perturbed Steps:

ID	Annotations Perturbed Before		Predictions Perturbed Before		Annotations Perturbed After		Predictions Perturbed After	
	**Diagonal**	**Frontal**	**Diagonal**	**Frontal**	**Diagonal**	**Frontal**	**Diagonal**	**Frontal**
ID1	69	69	61	30	69	69	65	62
ID2	64	64	56	53	64	64	57	55
ID3	95	95	16	3	95	95	46	33
ID4	64	64	53	22	64	64	54	41
ID5	79	79	30	11	79	79	46	38
Total Perturbed Steps	371	371	216	119	371	371	268	229

Appendix 4: Total steps (normal & perturbed steps)

Annotations vs. Predictions (detected by the AI model):

	Annotations	Predictions	
**ID1 (before)**	diagonal	diagonal	frontal
IC Right	122	116	77
IC Left	121	114	16
FC Right	121	117	56
FC Left	120	118	114
**ID1 (after)**			
IC Right		119	120
IC Left		122	117
FC Right		122	118
FC Left		121	120
**ID2 (before)**		diagonal	frontal
IC Right	113	110	110
IC Left	112	110	110
FC Right	113	111	109
FC Left	112	112	109

**ID2 (after)**			
IC Right		110	110
IC Left		112	111
FC Right		111	110
FC Left		117	109
**ID3 (before)**		diagonal	frontal
IC Right	149	113	0
IC Left	151	8	1
FC Right	148	108	15
FC Left	150	132	6
**ID3 (after)**			
IC Right		141	34
IC Left		40	83
FC Right		137	130
FC Left		141	75
**ID4 (before)**		diagonal	frontal

IC Right	107	107	66
IC Left	107	107	19
FC Right	108	105	87
FC Left	106	106	101
**ID4 (after)**			
IC Right		108	104
IC Left		117	105
FC Right		107	104
FC Left		108	107
**ID5 (before)**		diagonal	frontal
IC Right	129	101	40
IC Left	129	94	41
FC Right	129	106	87
FC Left	130	114	82
**ID5 (after)**			
IC Right		123	130
IC Left		121	110

FC Right		119	128
FC Left		124	115
**Total steps before:**		diagonal	frontal
IC Right	620	547	293
IC Left	620	433	187
FC Right	619	547	354
FC Left	618	582	412
Total:	2477 steps annotated	2109	1246
**Total steps after :**			
IC Right		601	498
IC Left		512	526
FC Right		596	590
FC Left		611	526
Total:		2320	2140
