# Supplementary material for: Validation of AI-based markerless gait event detection during perturbed walking using smartphone videos from two camera perspectives
Source: Front Sports Act Living. 2026 Aug 4;8:1902010. doi: 10.3389/fspor.2026.1902010 (PMC13481715; doi:10.3389/fspor.2026.1902010)

ID4_Frontal_before training

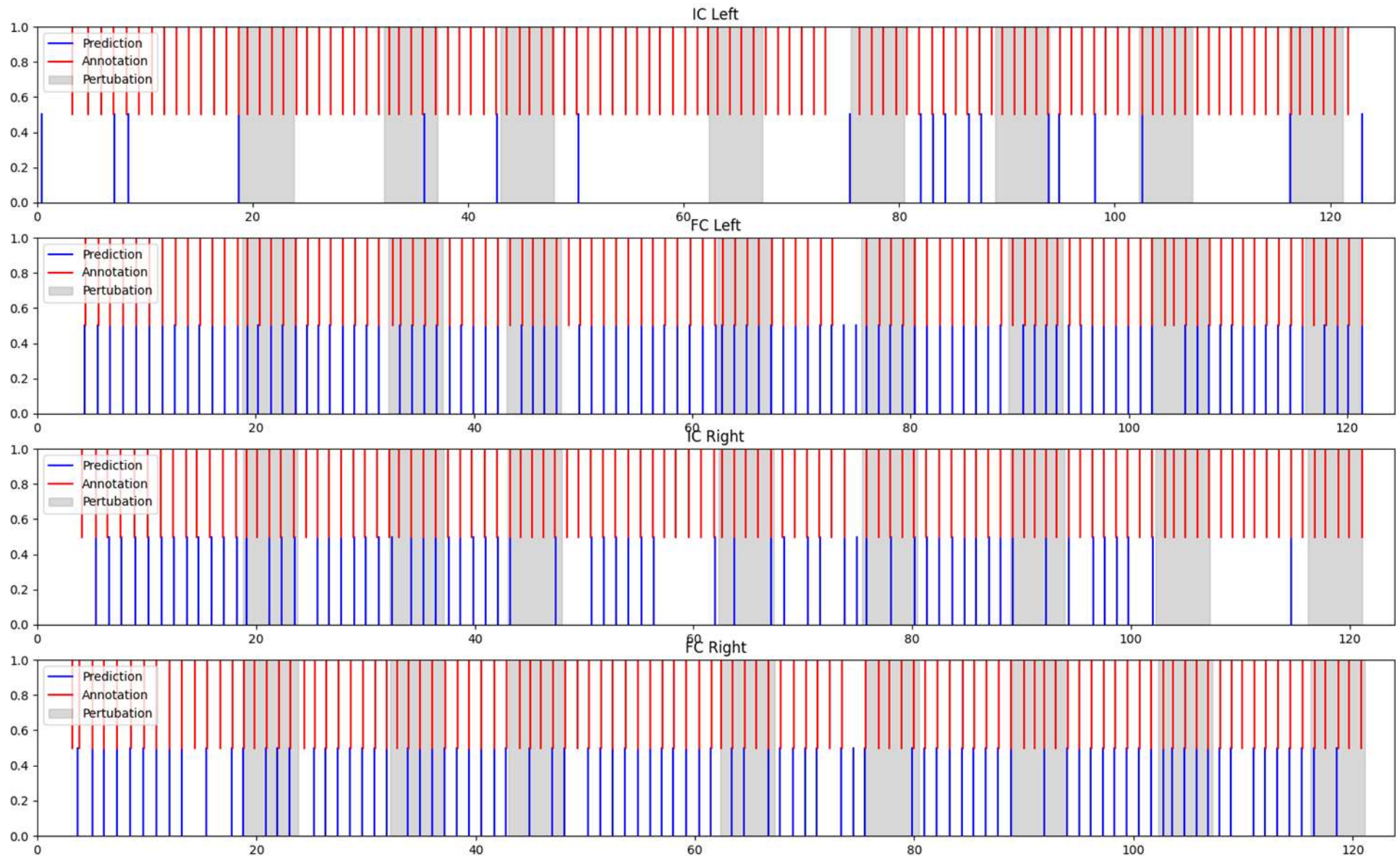

ID4_Frontal_after training

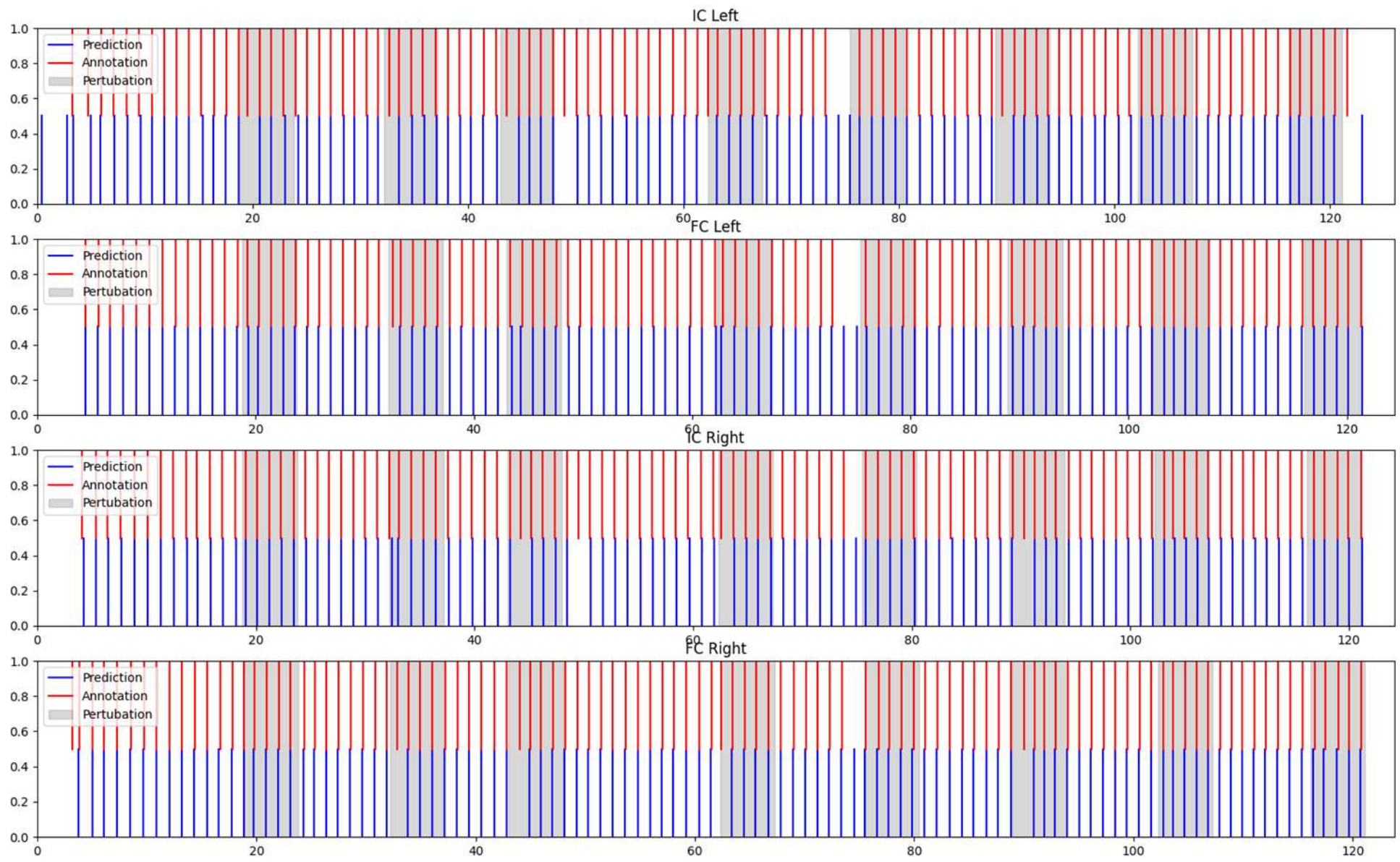

ID4_Diagonal_before training

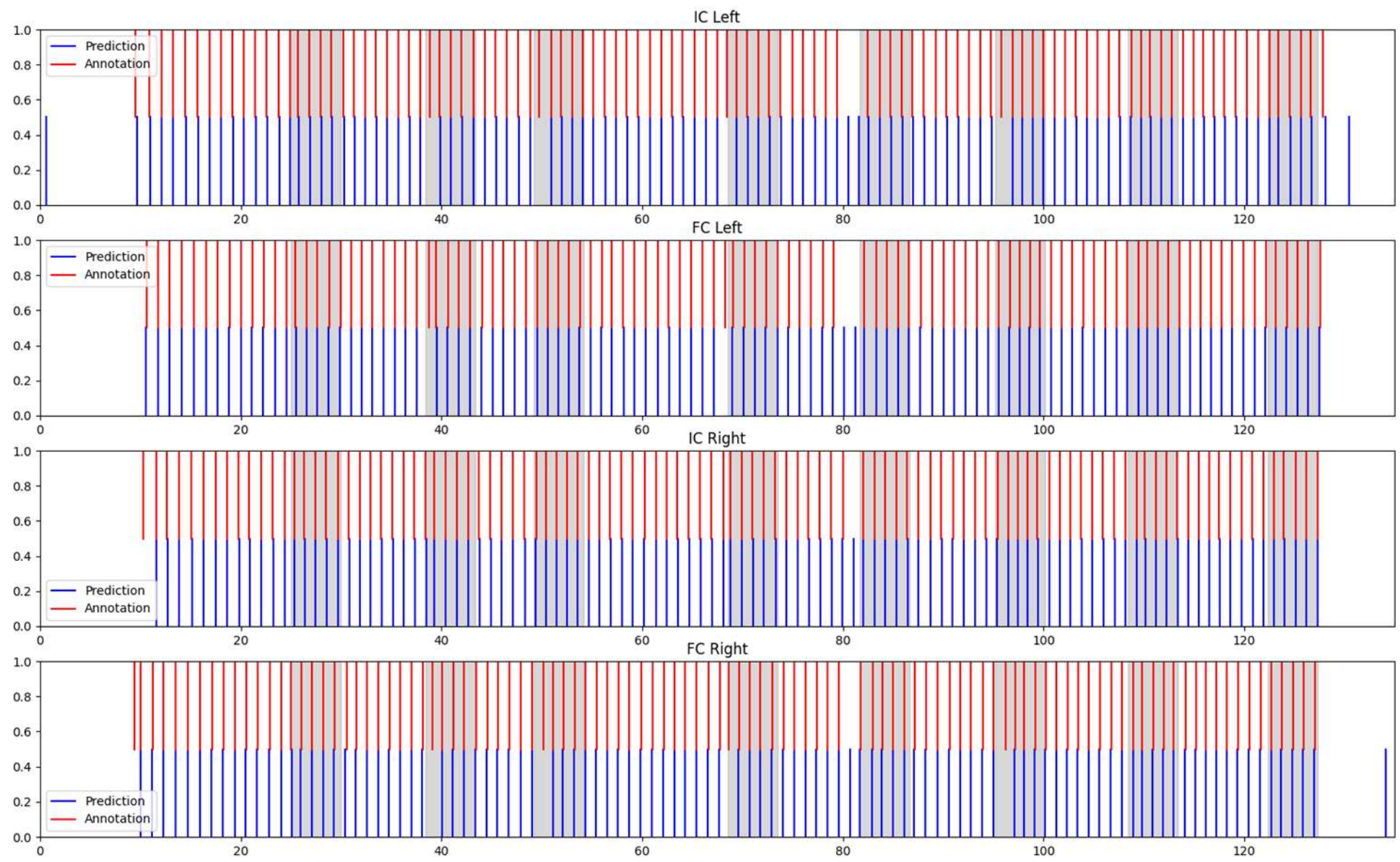

ID4_Diagonal_after training

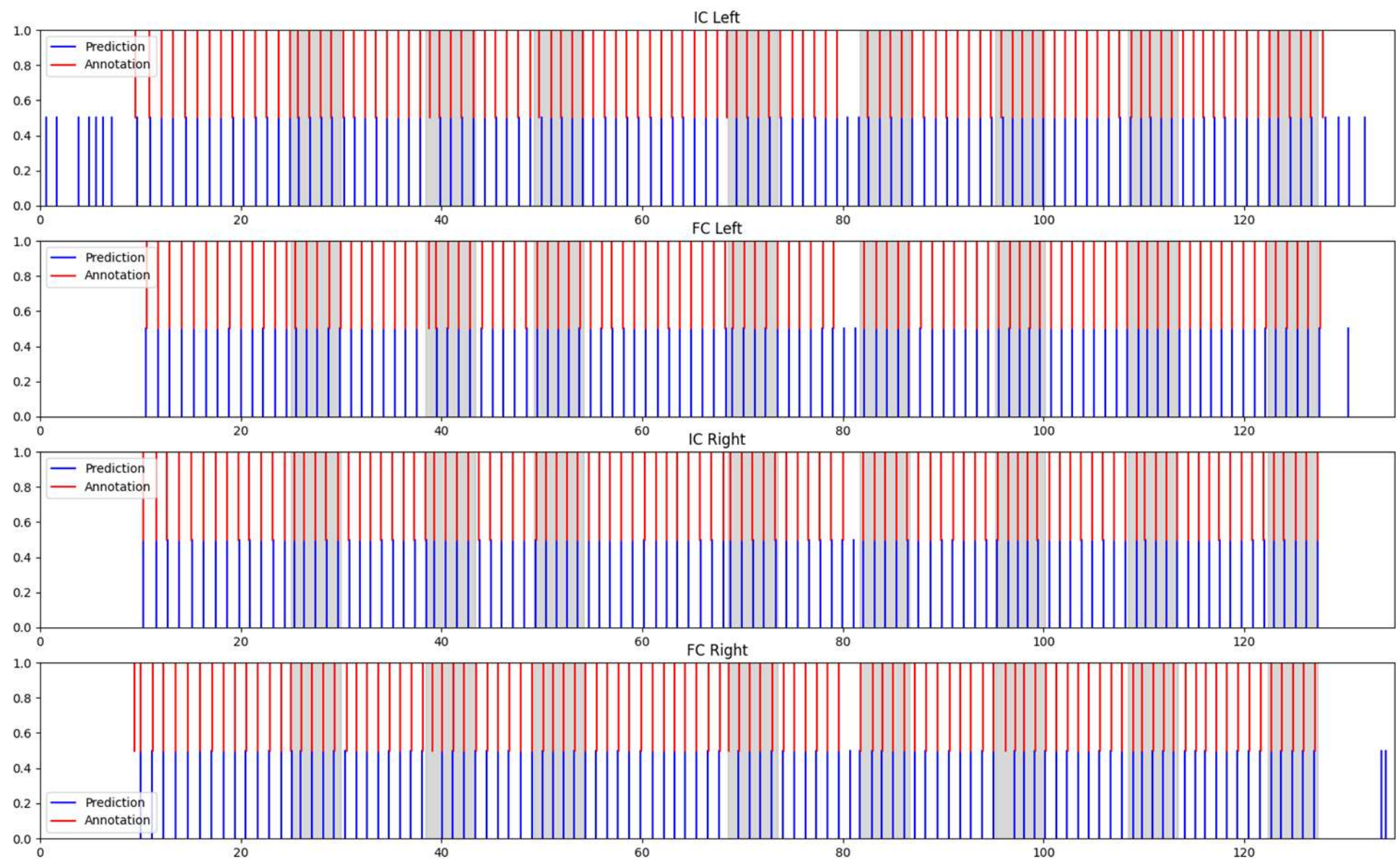

Supplement: Supplementary file 3 [file Datasheet3.pdf]
